# Public versus Private Healthcare Systems following Discharge from the ICU: A Propensity Score-Matched Comparison of Outcomes

**DOI:** 10.1155/2016/6568531

**Published:** 2016-03-30

**Authors:** Felippe Leopoldo Dexheimer Neto, Regis Goulart Rosa, Bruno Achutti Duso, Jaqueline Sanguiogo Haas, Augusto Savi, Cláudia da Rocha Cabral, Juçara Gasparetto Maccari, Roselaine Pinheiro de Oliveira, Ana Carolina Peçanha Antônio, Priscylla de Souza Castro, Cassiano Teixeira

**Affiliations:** Department of Critical Care, Hospital Moinhos de Vento, Ramiro Barcelos 910/605, 90035-001 Porto Alegre, RS, Brazil

## Abstract

*Purpose.* The long-term outcomes of patients after discharge from tertiary ICUs as they relate to the public versus private healthcare systems in Brazil have not yet been evaluated.* Materials and Methods.* A multicenter prospective cohort study was conducted to compare the all-cause mortality and the physical functional status (PFS) 24 months after discharge from the ICU between adult patients treated in the public and private healthcare systems. A propensity score- (PS-) matched comparison of all causes of mortality and PFS 24 months after discharge from the ICU was performed.* Results.* In total, 928 patients were discharged from the ICU including 172 (18.6%) patients in the public and 756 (81.4%) patients in the private healthcare system. The results of the PS-matched comparison of all-cause mortality revealed higher mortality rates among the patients of the public healthcare system compared to those of the private healthcare system (47.3% versus 27.6%, *P* = 0.003). The comparison of the PS-matched Karnofsky performance and Lawton activities of daily living scores between the ICU survivors of the public and private healthcare systems revealed no significant differences.* Conclusions.* The patients of private healthcare system exhibited significantly greater survival rates than the patients of the public healthcare system with similar PFS following ICU discharge.

## 1. Introduction

In Brazil, the federal legislation follows the principle that healthcare is a fundamental right that is inherent to every human being and that the state itself should provide the conditions to fully accomplish this goal (constitutional law 8.080, from September 19, 1990) [1]. Thereby, healthcare attention can be provided through the state and free of charge via the public healthcare system (*Sistema Único de Saúde*) or via health plans and/or by personal resources by the private healthcare system. Nevertheless, the deficiencies of the public healthcare system are notorious and broadly covered by the media. The struggles of assessing preventive measures and inpatient management can negatively weigh against the different stages of the health-sickness process of our population. In cases of severe illness in which the timing of the establishment of effective treatment is crucial, the consequences of delays can be devastating [2, 3].

According to the most recent census by the* Associação de Medicina Intensiva Brasileira* [4], the scope of intensive care in Brazil comprises 1.3 intensive care unit (ICU) beds for every 10 thousand people; this coverage is considered adequate by the Ministry of Health (Regulation number 1101/GM from June 12, 2002). However, little data is available about the indicators and performances of our ICUs, especially regarding patient outcomes after hospital discharge.

To confront this issue, the objective of the present study was to evaluate the long-term outcomes of patients who were discharged from ICUs and to compare the evolutions of those outcomes according to whether the care was provided by the public or private healthcare system.

## 2. Materials and Methods

### 2.1. Study Design, Setting, and Patients

A prospective cohort study was conducted in mixed medical/surgical ICUs from two tertiary referral hospitals in southern Brazil between July 2010 and July 2011. Both hospitals treated patients from the public and private healthcare systems during the study period. All adult patients (age > 18 years) who required admission to an ICU for more than 24 hours were followed up for 24 months after hospital discharge. The patients who did not survive the ICU stay were excluded from this analysis.

### 2.2. Independent Variables

The main independent variable in the present study was the healthcare source, that is, public or private. The public healthcare group was composed of patients for whom the only source of healthcare delivery was the* Sistema Único de Saúde*. The private healthcare group was composed of patients who paid the costs of hospitalization with health plans or personal resources.

The covariates analyzed in the present study included age, gender, number of comorbidities (i.e., heart failure, ischemic heart disease, cerebrovascular disease, diabetes mellitus, chronic obstructive pulmonary disease, cirrhosis, HIV infection, chronic renal failure, and malignant neoplasia), the ICU admission acute physiology and chronic health evaluation-II score (APACHE-II), requirements of mechanical ventilation and renal replacement therapy (RRT) during the ICU stay, the day of ICU discharge sequential organic failure assessment (SOFA) score, and the day of discharge simplified therapeutic intervention scoring system (TISS-28) score. The APACHE-II is a common scoring system that is used to grade the severity of illness in critically ill patients. The APACHE-II generates a point score in the range of 0 to 71 based on 12 physiologic variables, age, and underlying health. Higher scores indicate more severe acute illness [5]. The SOFA score is based on the extent of the patient's organic function as determined by physiological parameters of the respiratory, neurologic, cardiovascular, hepatic, coagulation, and renal systems. Higher scores indicate greater numbers of organic dysfunctions [6]. The TISS-28 score comprises interventions related to basic activities, ventilator support, cardiovascular support, renal support, neurologic support, metabolic support, and specific interventions. Higher scores indicate a patient's need to receive greater numbers of interventions [7].

### 2.3. Outcomes and Follow-Up

The primary outcome of the cohort was the all-cause mortality 24 months after discharge from the ICU. The secondary endpoint was the physical functional status (PFS) 24 months after discharge from the ICU. The grades of the PFSs among the ICU survivors were evaluated based on the Karnofsky performance score and the Lawton activities of daily living (ADL) score 24 months after discharge from the ICU. The Karnofsky performance score is a validated score that quantifies general well-being and the activities of daily life. The Karnofsky score ranges from 0 to 100; 0 indicates death, and 100 indicates perfect health [8]. The Lawton ADL score is an appropriated instrument that is used to assess independent living skills such as telephone use, shopping, food preparation, laundry, mode of transportation, the patients' responsibility for their own medications, and ability to handle finances [9]. The Lawton ADL score ranges from 0 to 32, and a higher score indicates greater ability levels.

The patients were followed up during their ICU stay by researchers who were not associated with the attending physician's team. With the aim of evaluating the study outcomes, follow-up phone calls were made 24 months after discharge from the ICU to all patients who survived their ICU stay. If a patient was deceased at the time of the phone call, the survival time was calculated based on the date of death reported by the proxy. The Karnofsky performance and Lawton ADL instruments were applied by phone by trained researchers. If the patient was unable to complete the phone interview, the questions were answered by a proxy; this proxy was the same person who provided information during the ICU stay when possible. Periodic evaluations were performed to determine the interrater reliability and to ensure that the quality of the interviews remained similar between the data collectors.

### 2.4. Statistical Analysis

Observational studies are often limited by imbalances in both known and unknown confounders; here, such confounders might have caused some patients who were discharged from ICUs in the public healthcare system to be more likely to develop unfavorable long-term outcomes compared to the patients in the private healthcare system. Therefore, we applied propensity score (PS) matching to balance the baseline characteristics and reduce the probability of selection bias [10]. The PS (probability of being treated in the public healthcare system) was calculated using a stepwise multivariate logistic regression model in which the dependent variable was treatment in the public healthcare system. All variables that could potentially have influenced the probability of being treated in the public healthcare system and had a *P* value < 0.20 in a univariate analysis were included. In the multivariate model, the independent variables were eliminated from the highest to the lowest *P* value but were retained in the model if the *P* value was <0.10 (backward method). Matching was performed via the use of a 1 : 1 matching protocol without replacement (nearest neighbor algorithm). Standardized differences were estimated for all the baseline covariates before and after matching to assess the prematch imbalance and the postmatch balance. Standardized differences ≤ 10.0% for a given covariate indicated relatively small imbalances. In the matched cohort, paired comparisons were performed with McNemar's tests for binary variables and paired Student's *t*-tests for continuous variables. Kaplan-Meier curves were used to calculate the time-dependent occurrence of death in the matched pairs to preserve the benefit of the matching. The log-rank test was used for comparisons between groups. A significance level of 0.05 was adopted for all statistical comparisons. The software used for the statistical analysis was STATA version 12 (StataCorp LP, TX, USA).

### 2.5. Ethical Issues

Written informed consent was obtained from all study participants on the day of discharge from ICU. The institutional review board of the Moinhos de Vento Hospital and Complexo Hospitalar Santa Casa de Misericórdia de Porto Alegre approved the study.

## 3. Results

**Figure 1:**
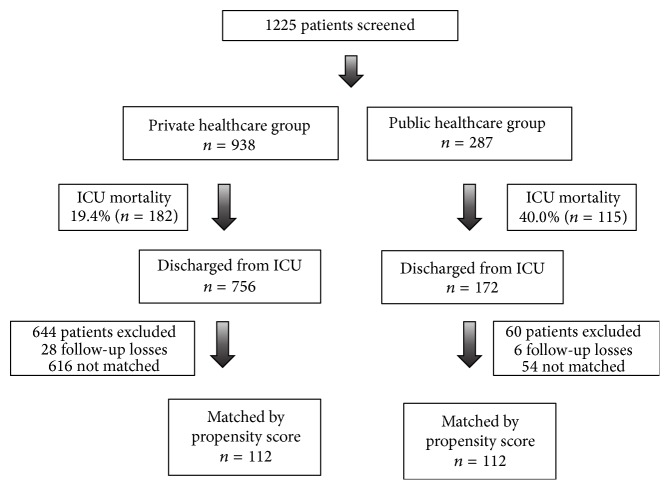
Study population. ICU: intensive care unit.

During the study period, 1225 patients were evaluated (Figure 1). Of these, 928 patients were discharged from an ICU; 172 (18.6%) of these patients were in the public healthcare system, and 756 (81.4%) of the patients were in the private healthcare system. Loss to follow-up occurred in 34 patients (6 patients [3.4%] in the public healthcare system and 28 patients [3.7%] in the private healthcare system). After PS matching, 112 pairs of patients were identified. The overall mortality of the study population 2 years after discharge from the ICU was 37.5% (84 deaths). Among the survivors, the mean Karnofsky performance and Lawton ADL scores were 79.2 (standard deviation [SD] ± 17.5) and 24.6 (SD ± 10.2), respectively.

**Figure 2:**
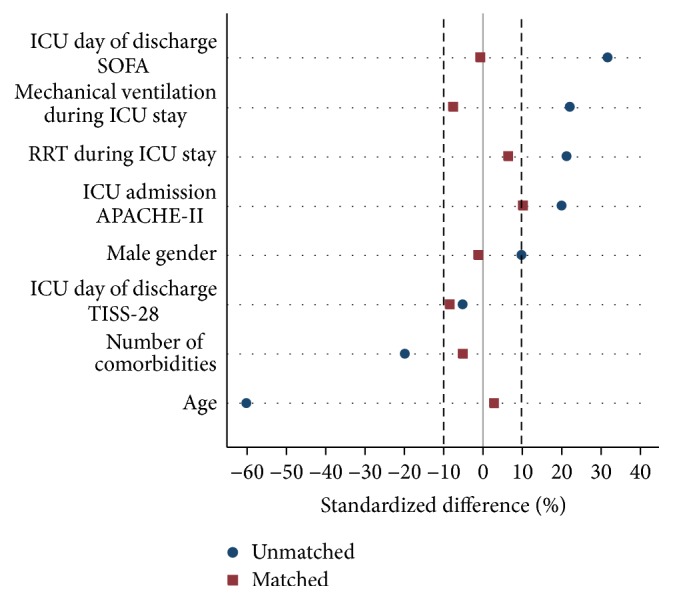
Balances of the covariates in the public and private healthcare systems before and after propensity score matching.* Note.* After propensity score matching, 112 matched pairs were identified. The standardized differences are reported as percentages, and differences ≤ 10.0% indicate relatively small imbalances. ICU: intensive care unit; APACHE-II: acute physiology and chronic health evaluation-II score; SOFA: sequential organ failure assessment score; TISS-28: simplified therapeutic intervention scoring system; RRT: renal replacement therapy.

The baseline clinical characteristics of all patients evaluated in the present cohort are shown in Table 1. Due to the nonrandomized design, the baseline characteristics of the patients discharged from the ICU in the private healthcare system differed from those of the patients discharged from the public healthcare system. These differences were particularly important in terms of age, the number of comorbidities, ICU admission APACHE-II score, mechanical ventilation and RRT during the ICU stay, and SOFA score of the day of ICU discharge. However, after PS matching, all of these differences decreased to nonsignificant levels, which suggests that the PS matching appropriately adjusted for the initial treatment selection bias (Figure 2).

**Figure 3:**
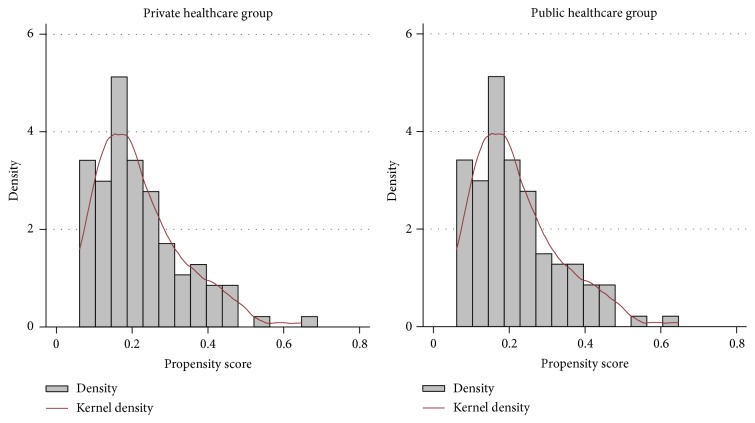
Distributions of the propensity scores of the critical care patients according to healthcare status after propensity matching.

Table 2 illustrates the multivariate logistic regression analysis of the factors associated with treatment in the public healthcare system. Younger patients and patients with higher ICU admission APACHE-II and ICU day of discharge SOFA scores were more likely to be treated in the public healthcare system. Additionally, the needs for mechanical ventilation and RRT during the ICU stay were greater in the patients who were treated in the public healthcare system. The results of this logistic regression model were used to build the PS. The distribution of PSs according to healthcare status after propensity score matching is displayed in Figure 3.

**Figure 4:**
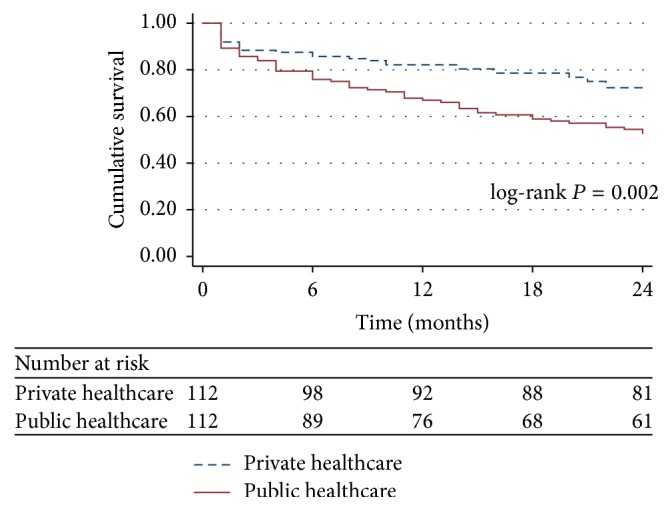
Survival curves of the critical care patients discharged from tertiary ICUs according to healthcare system status: propensity score-matched analysis. ICU: intensive care unit.

The comparison of the PS-matched all-cause mortalities revealed a higher mortality rate among the patients in the public healthcare system compared to those in the private healthcare system (47.3% versus 27.6%, resp., *P* = 0.003; Table 3). The comparison of the PS-matched survival curves between the patients in the public and private healthcare systems is illustrated in Figure 4. The amplitude of difference in the survival rates according to healthcare system status increased within the first 18 months after discharge from the ICU and remained constant after this period (log-rank *P* = 0.002).

**Figure 5:**
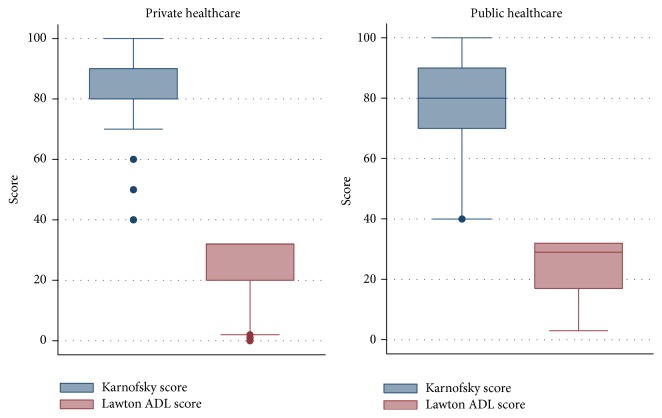
Comparison of the physical functional status scores of the ICU survivors according to healthcare system status.* Note.* Comparisons of the Karnofsky and Lawton ADL scores among survivors 24 months after discharge from the ICU (there were 81 patients in the private healthcare group and 59 patients in the public healthcare group). There were no significant differences between the two groups of patients. ICU: intensive care unit; ADL: activity of daily living.

Among the survivors 24 months after discharge from the ICU (81 patients in the private healthcare group and 59 patients in the public healthcare group), the Karnofsky performance and Lawton ADL scores were statistically similar between both the private and public healthcare system groups (Figure 5). The proportions of patients with Karnofsky performance scores ≤50 points were 12.3% (10 patients) in the private healthcare group and 11.8% (7 patients) in the public healthcare group. The proportions of patients with Lawton ADL scores ≤16 points were 18.5% (15 patients) in the private healthcare group and 20.3% (12 patients) in the public healthcare group.

## 4. Discussion

The present prospective cohort revealed that the patients in the public healthcare system exhibited significantly greater mortality rates than the patients in the private healthcare system after ICU discharge. Despite the lower mortality rates, the patients in the private healthcare system exhibited PFSs that were similar to those of the patients of public healthcare system.

Similar to our findings, Nicolau et al. [11] compared the mortality rates during and after hospital admission in patients with acute myocardial infarction who benefited from the private or the public healthcare system. These authors demonstrated that the public healthcare patients exhibited the same in-hospital mortality rate (10.3% versus 11.4%; *P* = 0.5) but also exhibited an increased chance of long-term mortality (36% higher odds; *P* = 0.005) compared with the private healthcare patients. Additionally, in the United States where access to healthcare is not universal, several studies have correlated intake and the quality of care directly with health insurance coverage [3, 12–17]. A retrospective population analysis demonstrated that patients with myocardial infarction and pneumonia and without health plans exhibited a higher rate of in-hospital mortality [12]. Similarly, Trinh et al. [13] evaluated the postoperative evolution of 61167 radical prostatectomies, compared them according to the payer source, and observed better outcomes among the patients with private health insurance plans. Together, these findings suggest an inverse correlation of mortality with the socioeconomic conditions (SEC) of enrolled patients and a direct correlation with disease severity at the time of diagnosis [18, 19]. The SEC is categorized according to education, occupation, income, and availability of both health and cultural resources [18]. Patients who use the public healthcare system are more likely to have less education and income. These factors, in association with a poor organizational health structure, could limit the access of ICU survivors to healthcare [11]. An Australian cohort of 15619 critically ill patients demonstrated that those with the worst SEC were also younger and had more severe conditions and that the long-term mortality among this group was also higher despite the in-hospital mortality of these patients being similar to the patients with better SEC [19]. The findings of greater numbers of deaths after ICU discharge in the public healthcare patients throughout our study corroborate this idea. It is possible that mortality after discharge would have been comparable between the two groups if there were an equivalent number of readmissions; however, the present study does not allow us to reach further conclusions.

Brazil has a structure for healthcare that basically focuses on primary attention. The baseline of public healthcare provision occurs within local governments' ability to first evaluate patients and, whenever necessary, refer them to higher levels of care. The reliance on community health centers and medical teams to assist families is strongly present in the national territory through the idea of gatekeeping and referral [20]. This process of patient flow is mandatory and aims mainly to optimize costs in a context in which the public health funding is not sufficient to meet the demands of population [21]. Nevertheless, this model of care has limitations especially when the process of referral is disorganized and patient is complex and extremely dependent on multidisciplinary rehabilitation care, as well as the postintensive care patient [22]. While the public healthcare system faces obstacles related to bureaucracy, lack of specific guidelines to manage patients after ICU discharge, and delay during the referral process of the postintensive care patient, the private healthcare system allows faster access to medical subspecialties and specialized rehabilitation care. These peculiarities may explain, at least in part, the differences in the survival rates after ICU discharge between adult patients treated in the public and private healthcare systems.

In the present study, two scales were used to assess the PFS with the goal of increasing the reliability of our results. Studies assessing the quality of life after ICU discharge suggest that these patients do not return to the same level of health that they had before they fell ill and that their quality of life is lower than that of the general population, at least in the early years [23]. Interestingly, our data revealed that the source of care, that is, public or private, did not influence the PFSs of the survivors. However, our results should be interpreted with caution because a higher proportion of survivors (and probably sicker patients) were present in the private healthcare group.

The present study has some limitations. The intensity and quality of care after ICU discharge, direct SEC variables, and rehospitalization rates were not examined. Furthermore, this study was susceptible to the biases that are inherent in observational studies (e.g., confounding factors caused by unmeasured variables that were not balanced by the PS matching procedure); however, the possibility of systematic errors was minimized by the proper measurement of variables and outcomes with previously defined objective criteria, the use of standardized data collection, a follow-up that was performed by a research team that was not involved in patient care, and the application of PS matching, which allowed for the balancing of important covariates in the two study arms.

## 5. Conclusions

The patients in the public healthcare system exhibited significantly greater mortality rates than the patients in the private healthcare system following ICU discharge. Despite the lower mortality rates, the patients in the private healthcare system exhibited PFSs that were similar to those of the patients in the public healthcare system. These results may be explained by differences in the quality of post-ICU care between the patients in the public and private healthcare systems.

## Figures and Tables

**Table 1:** Comparison of baseline characteristics of critical care patients discharged from tertiary hospitals according to healthcare system status.

Variables	All cohort (*n* = 928)	Private healthcare group (*n* = 756)	Public healthcare group (*n* = 172)	*P* value
Male gender, *n* (%)	499 (53.7)	401 (53.0)	98 (56.9)	0.39
Age, years, mean ± SD	63.7 ± 17.6	65.7 ± 17.3	55.3 ± 16.7	<0.001
Number of comorbidities^*∗*^, mean ± SD	1.4 ± 1.2	1.5 ± 1.2	1.2 ± 1.1	0.02
ICU admission APACHE-II, mean ± SD	14.9 ± 6.6	14.7 ± 6.6	15.9 ± 6.6	0.03
Mechanical ventilation during ICU stay, *n* (%)	324 (34.9)	248 (32.8)	76 (40.9)	0.006
RRT during ICU stay, *n* (%)	67 (7.2)	46 (6.0)	21 (12.2)	0.008
ICU day of discharge SOFA score, mean ± SD	0.8 ± 1.6	0.7 ± 1.5	1.28 ± 1.8	<0.001
ICU day of discharge TISS-28 score, mean ± SD	11.6 ± 4.6	11.6 ± 4.5	11.4 ± 4.9	0.16

*Note*. ^*∗*^The comorbidities included heart failure, ischemic heart disease, cerebrovascular disease, diabetes mellitus, chronic obstructive pulmonary disease, cirrhosis, HIV infection, chronic renal failure, and malignant neoplasia.

SD: standard deviation; ICU: intensive care unit; APACHE-II: acute physiology and chronic health evaluation-II score; RRT: renal replacement therapy; SOFA: sequential organ failure assessment score; TISS-28: simplified therapeutic intervention scoring system.

**Table 2:** Multivariate logistic regression of the factors associated with care in the public healthcare system: propensity score model.

Variable	OR	95% CI	*P* value
Age, per year	0.96	0.95–0.97	<0.001
ICU admission APACHE-II, per point	1.02	0.99–1.05	0.05
Mechanical ventilation during ICU stay	1.47	1.00–2.16	0.04
ICU day of discharge SOFA, per point	1.19	1.08–1.32	<0.001
ICU day of discharge TISS-28, per point	0.96	0.92–1.00	0.05

*Note*. The following variables were entered into the model: age, number of comorbidities, ICU admission APACHE-II, mechanical ventilation during ICU stay, RRT during ICU stay, ICU day of discharge SOFA, and ICU day of discharge TISS-28.

OR: odds ratio; CI: confidence interval; ICU: intensive care unit; APACHE-II: acute physiology and chronic health evaluation-II score; SOFA: sequential organ failure assessment score; TISS-28: simplified therapeutic intervention scoring system; RRT: renal replacement therapy.

**Table 3:** All-cause mortality in the critical care patients 24 months after discharge from the ICU according to healthcare system status: propensity score-matched analysis.

	Private healthcare group (*n* = 112)	Public healthcare group (*n* = 112)	*P* value
Mortality rate, *n* (%)	31 (27.6)	53 (47.3)	0.003

## Abbreviations

AMIB: Associação de Medicina Intensiva Brasileira
APACHE-II: Acute physiology and chronic health evaluation-II score
ADL: Activities of daily living
CI: Confidence interval
ICU: Intensive care unit
OR: Odds ratio
PFS: Physical functional status
PS: Propensity score
RRT: Renal replacement therapy
SEC: Socioeconomic conditions
SD: Standard deviation
SOFA: Sequential organic failure assessment score
TISS-28: Simplified therapeutic intervention scoring system.

## References

[1] Legislação do SUS http://bvsms.saude.gov.br/bvs/publicacoes/progestores/leg_sus.pdf.

[2] Danis M., Linde-Zwirble W. T., Astor A., Lidicker J. R., Angus D. C. (2006). How does lack of insurance affect use of intensive care? A population-based study. *Critical Care Medicine*.

[3] Fowler R. A., Noyahr L.-A., Thornton J. D. (2010). An official American Thoracic Society systematic review: the association between health insurance status and access, care delivery, and outcomes for patients who are critically ill. *American Journal of Respiratory and Critical Care Medicine*.

[4] Censo AMIB http://www.amib.org.br/fileadmin/CensoAMIB2010.pdf.

[5] Breslow M. J. (2012). Severity scoring in the critically ill: part 1—interpretation and accuracy of outcome prediction scoring systems. *Chest*.

[6] Vincent J. L., Moreno R., Takala J. (1996). The SOFA (Sepsis-related Organ Failure Assessment) score to describe organ dysfunction/failure: on behalf of the Working Group on Sepsis-Related Problems of the European Society of Intensive Care Medicine. *Intensive Care Medicine*.

[7] Moreno R., Morais P. (1997). Validation of the simplified therapeutic intervention scoring system on an independent database. *Intensive Care Medicine*.

[8] Karnofsky D. A., Burchenal J. H., MacLeod C. M. (1949). Evaluation of chemotherapeutic agents. *The Clinical Evaluation of Chemotherapeutic Agents in Cancer*.

[9] Graf C. (2008). The lawton instrumental activities of daily living scale. *American Journal of Nursing*.

[10] Martens E. P., Pestman W. R., de Boer A., Belitser S. V., Klungel O. H. (2008). Systematic differences in treatment effect estimates between propensity score methods and logistic regression. *International Journal of Epidemiology*.

[11] Nicolau J. C., Baracioli L. M., Serrano C. V. (2008). The influence of health insurance plans on the long term outcome of patients with acute myocardial infarction. *Arquivos Brasileiros de Cardiologia*.

[12] Hasan O., Orav E. J., Hicks L. S. (2010). Insurance status and hospital care for myocardial infarction, stroke, and pneumonia. *Journal of Hospital Medicine*.

[13] Trinh Q.-D., Schmitges J., Sun M. (2012). Morbidity and mortality of radical prostatectomy differs by insurance status. *Cancer*.

[14] Kahn K. L., Pearson M. L., Harrison E. R. (1994). Health care for black and poor hospitalized Medicare patients. *The Journal of the American Medical Association*.

[15] Jencks S. F., Cuerdon T., Burwen D. R. (2000). Quality of medical care delivered to medicare beneficiaries: a profile at state and national levels. *The Journal of the American Medical Association*.

[16] Moonesinghe R., Chang M. H., Truman B. I. (2013). Health insurance coverage—United States, 2008 and 2010. *MMWR Surveillance Summaries*.

[17] Schoenfeld A. J., Belmont P. J., See A. A., Bader J. O., Bono C. M. (2013). Patient demographics, insurance status, race, and ethnicity as predictors of morbidity and mortality after spine trauma: a study using the National Trauma Data Bank. *Spine Journal*.

[18] Bein T., Hackner K., Zou T. (2012). Socioeconomic status, severity of disease and level of family members' care in adult surgical intensive care patients: the prospective ECSSTASI study. *Intensive Care Medicine*.

[19] Ho K. M., Dobb G. J., Knuiman M., Finn J., Webb S. A. (2008). The effect of socioeconomic status on outcomes for seriously ill patients: a linked data cohort study. *Medical Journal of Australia*.

[20] Franks P., Clancy C. M., Nutting P. A. (1992). Gatekeeping revisited—protecting patients from overtreatment. *The New England Journal of Medicine*.

[21] Cardoso F. (2014). Health in Brazil—optmistic outlook?. *Arquivos Brasileiros de Cirurgia Digestiva*.

[22] Willems D. L. (2001). Balancing rationalities: gatekeeping in health care. *Journal of Medical Ethics*.

[23] Oeyen S. G., Vandijck D. M., Benoit D. D., Annemans L., Decruyenaere J. M. (2010). Quality of life after intensive care: a systematic review of the literature. *Critical Care Medicine*.

