# Corrigendum to “Public versus Private Healthcare Systems following Discharge from the ICU: A Propensity Score-Matched Comparison of Outcomes”

**DOI:** 10.1155/2018/8751513

**Published:** 2018-09-06

**Authors:** Felippe Leopoldo Dexheimer Neto, Regis Goulart Rosa, Bruno Achutti Duso, Jaqueline Sangiogo Haas, Augusto Savi, Cláudia da Rocha Cabral, Juçara Gasparetto Maccari, Roselaine Pinheiro de Oliveira, Ana Carolina Peçanha Antônio, Priscylla de Souza Castro, Cassiano Teixeira

**Affiliations:** Department of Critical Care, Hospital Moinhos de Vento, Ramiro Barcelos 910/605, 90035-001 Porto Alegre, RS, Brazil

In the article titled “Public versus Private Healthcare Systems following Discharge from the ICU: A Propensity Score-Matched Comparison of Outcomes” [1], the name of the fourth author was given incorrectly as Jaqueline Sanguiogo Haas. The author's name should have been written as Jaqueline Sangiogo Haas. The revised authors' list is shown above.
